# Performance of a score to characterise adequate contact among the social network of persons with TB

**DOI:** 10.5588/ijtldopen.24.0376

**Published:** 2024-12-01

**Authors:** M.E. Castellanos, S. Zalwango, T.H.T. Quach, R. Kakaire, L. Martínez, M.H. Ebell, K.K. Dobbin, N. Kiwanuka, C.C. Whalen

**Affiliations:** ^1^ Global Health Institute, College of Public Health, University of Georgia, Athens, GA, USA;; ^2^ Department of Epidemiology and Biostatistics, College of Public Health, University of Georgia, Athens, GA, USA;; ^3^ Public Health and Tropical Medicine, College of Public Health, Medical and Veterinary Sciences, James Cook University, Townsville, QLD, Australia;; ^4^ Makerere University College of Health Sciences, School of Public Health, Kampala, Uganda;; ^5^ Division of Infectious Diseases and Geographic Medicine, Stanford University School of Medicine, Stanford, CA, USA;; ^6^ Department of Epidemiology, School of Public Health, Boston University, Boston, MA, USA.

**Keywords:** tuberculous infection, tuberculosis, contact, transmission, social network

## Abstract

**BACKGROUND:**

Transmission of *Mycobacterium tuberculosis* requires adequate contact between an infectious case and a susceptible host. The aim of this analysis was to validate a recently developed contact score that assessed settings of exposure and relationships between the case and contact.

**METHODS:**

In a cross-sectional study from Kampala, Uganda, we estimated the prevalence of tuberculous infection (TBI) in social contacts of adult TB cases according to the setting and relationship domains of the contact score. We calculated the prevalence ratio (PR) for the association between contact scores (by domain) with TBI using modified Poisson regression models.

**RESULTS:**

We enrolled 955 household and community contacts from 119 TB cases. The prevalence of TBI in the social network was 52% (95% CI 48–55). The prevalence of TBI increased by quartile for both the setting score (44%, 40%, 53%, 70%; *P*_trend_ <0.0001) and the relationship score (41%, 47%, 53%, 66%; *P*_trend_ <0.0001). The setting score was associated with a higher prevalence of infection among children aged 5–14 years, whereas the relationship score was associated with infection in children aged 0–4 years.

**CONCLUSION:**

In urban Africa, contacts of TB with higher settings and relationship scores were more likely infected with *M. tuberculosis*.

## RÉSUMÉ

**CONTEXTE:**

La transmission de *Mycobacterium tuberculosis* nécessite un contact adéquat entre un cas infectieux et un hôte réceptif. Cette étude visait à valider un score de contact récemment élaboré, destiné à évaluer les contextes d'exposition ainsi que les interactions entre le cas et le contact.

**MÉTHODES:**

Dans une étude transversale réalisée à Kampala, en Ouganda, nous avons évalué la prévalence de l'infection tuberculeuse (TBI) parmi les contacts sociaux des cas de TB adultes, en prenant en considération les divers aspects du cadre ainsi que leur lien avec le score de contact. Nous avons calculé le rapport de prévalence (PR) afin d'analyser la relation entre les scores de contact (selon les différents cadres) et la TBI, en recourant à des modèles de régression de Poisson ajustés.

**RÉSULTATS:**

Nous avons recruté 955 contacts au sein des ménages et des communautés liés à 119 cas de TB. La prévalence du TBI dans le cercle social s'élevait à 52% (IC à 95% 48–55). Cette prévalence a montré une augmentation par quartile en fonction du score d'établissement (44%, 40%, 53%, 70%, respectivement ; *P*_trend_ <0,0001) et du score de relation (41%, 47%, 53%, 66%, respectivement ; *P*_trend_ <0,0001). Le score d'établissement était corrélé à une prévalence plus élevée de l'infection chez les enfants de 5 à 14 ans, tandis que le score de relation était lié à l'infection chez les enfants de 0 à 4 ans.

**CONCLUSION:**

Dans les zones urbaines d'Afrique, les contacts ayant des scores de cadre et de relation plus élevés étaient plus enclins à être infectés par *M. tuberculosis*.

TB remains an endemic disease in parts of Southeast Asia and Africa despite optimising treatment through directly observed therapy.^1^ TB persists in these regions because one infectious case is replaced by at least one other case over time.^2^ An essential measure in the control of TB is to interrupt these chains of transmission. To achieve this end, we need to better understand the nature of *Mycobacteria tuberculosis* transmission.

Household contact studies have provided a useful design for discovering factors from the index case or the contact that can increase the risk of TB transmission, as measured by tuberculous infection (TBI). The age of the contact, sputum smear grade of the index case, cavitary lung disease, and the number of people living in a household are just some of the factors that have been identified.^3–5^ These factors alone will not cause a transmission event unless adequate contact between the infectious case and a susceptible host exists. Previous studies have shown that the nature of the contact of an index case with a household contact can increase the likelihood of TB transmission.^6,7^ For example, spouses are at higher risk of TBI than other household members, and daily contact can increase the risk of this infection in the household.^8,9^

Less is known about the transmission of *M. tuberculosis* outside the household. From epidemiologic and molecular studies conducted in diverse settings, it appears that over 80% of *M. tuberculosis* transmission occurs outside the households of index cases.^3,10,11^ From anecdotal reports of community outbreaks of TB,^12–14^ we know that some congregate settings are associated with transmission and the risk for TB disease, but the nature of interactions between infectious cases and susceptible contacts that define adequate contact for transmission in these settings is still poorly understood. For this reason, there remains uncertainty about how best to implement public health strategies that detect and prevent TB transmission in community settings.

To better understand the nature of interactions that may lead to *M. tuberculosis* transmission in the community, we have used factor analysis and developed a contact score to characterise the nature of human interactions within social networks in an African city.^15^ The aim of this study is to determine whether this score covaries with the presence of TBI among these contacts. We hypothesise that higher scores correspond with more extensive exposure and, therefore, a higher probability of *M. tuberculosis* transmission.

## METHODS

### Study population

We enrolled TB index cases aged ≥15 years in Lubaga Division, Kampala, Uganda, from 2012 to 2016. Index cases were microbiologically confirmed by a positive sputum smear and had signs and symptoms of pulmonary TB. We then ascertained their social networks, including contacts within and outside the household. These contacts were traced and enrolled; their demographic and clinical information was collected using standardised interviews. More details of this study have been previously provided.^11,15^

### Derived risk score

The study exposure was a derived score between TB cases and their contacts. The development of this score has been previously described.^15^ Briefly, index cases answered questions about the social mixing between them and their social network members. A factor analysis was conducted among these variables, identifying two main domains. The setting domain comprised six variables: nature of ventilation at the usual place of meeting, frequency of sleeping in the same room/same bed, the most recent meeting was indoors or outdoors, frequency of shared meals since the onset of cough, place of usual meeting (home vs. other location) and frequency and duration of contact over the past month. The relationship domain also included six variables: case trusted contact, case shared TB diagnosis with contact, case was provided care by the contact in the past 3 months, length of knowing contact, how well the case knows the contact, means of transportation used most often with contact-none/walking vs. a type of transportation. Factor analysis results provided weights for each of these variables. We then took the sum of these weights to obtain a setting and a relationship score for each interaction case contact. We have previously shown that these domain scores have construct validity, as they are consistent with other variables used to measure the extent and nature of the contact between an infectious case and susceptible contact.^15^

### Outcome

The study outcome was TBI in contacts, either latent TBI (LTBI) or active disease. LTBI was determined using the tuberculin skin test (TST). A positive TST result was defined as an induration ≥10 mm, as it has shown to be an adequate cut-off in the Ugandan setting.^6^ Active TB was defined as the presence of at least one smear positive for acid-fast bacilli, positive culture for *M. tuberculosis*, positive molecular result for *M. tuberculosis*, and a history of previous TB disease, informed by the social contact. Contacts who did not meet the definitions for TBI were classified as uninfected.

### Analytical strategy

The main objective of this study was to estimate and compare the prevalence of TBI according to contact scores. In the analysis, we included contacts with complete exposure and outcome data. We created a matrix of infection prevalence according to setting and relationship score combinations. We rounded scores to the nearest integer for presentation. We explored the association of the 12 individual variables that comprised the setting and relationship factors with the prevalence of TBI in social contacts of TB cases. TBI prevalence according to each response is shown with 95% confidence intervals (CIs).

We categorised setting and relationship scores as very low, low, moderate, and high contact according to quartiles. We estimated the prevalence and 95% CIs of TBI in each category, overall and stratifying by location of exposure (household or extra-household) and smear grade. We used Cochran-Armitage tests to assess for trends across settings and relationship quartiles.

We used Poisson regression models with robust variance to estimate the prevalence ratio (PR) for the association between increasing scores in setting and relationship domains and TBI.^16–18^ This technique allows the analysis of clustered data.^19^ Final models included the setting or relationship score and pre-selected potential confounders: age, sex, HIV status, body mass index (BMI) and smear grade of the index case, and the age, sex, BCG, and HIV status of contacts.^6,20–27^ We also included independent social factors associated with TBI. The final models are presented stratified by age of the contact, as the stratified analysis and regression models suggested that age of contact was an effect modifier of the association. Age was categorised as 0–4 years, 5–14 years, and ≥15 years, All analyses were conducted using SAS software v 9.4 (SAS Institute, Cary, NC, USA) and R v3.6.0 (R Foundation for Statistical Computing, Vienna, Austria, 2016).

### Ethics approval and consent to participate

Written informed consent was obtained from all participants before study enrolment. Institutional review board clearance was obtained from the Higher Degrees Research and Ethics Committee at Makerere University School of Public Health, Kampala, Uganda, and approved by the Uganda National Council for Science and Technology, Kampala, Uganda and the Institutional Review Board at the University of Georgia, Athens, GA, USA.

## RESULTS

Contacts of 119 index cases were enrolled in the study. Complete data for this study was available for 955 of 1006 contacts (95%; Supplementary Figure S1). The median age of index cases was 28 years (interquartile range [IQR] 23–36), while 82% were men. Almost one in five index cases were living with HIV (17%). The median age of the contacts was 23 years (IQR 13–31), and 52% were women (Table 1). Almost two-thirds of contacts were from outside the household (61%). Sex assortment with cases differed by sex; one-third of female contacts were exposed to a female index case, whereas 73% of male contacts were exposed to a male index case.

**Table 1.** Characteristics of the enrolled contacts of TB cases from Kampala, Uganda, who answered the social network survey (*n* = 119).

Characteristic	*n* (%)
Contacts enrolled	1,006 (100)
Contacts enrolled with complete data	955 (95)
Sex	
Male	462 (48)
Female	493 (52)
Type of contact	
Household	369 (39)
Extra-household	586 (61)
Age, years, median [IQR]	23 [13–31]
Age group, years	
0–4	110 (12)
5–14	141 (15)
≥15	704 (74)
Place of residence	
Lives in Lubaga Division	920 (96)
Does not live in Lubaga Division	34 (4)
No information available	1 (0)
HIV result	
Positive	71 (7)
Negative	874 (92)
Not done/refusals	10 (1)
BCG vaccine	
Yes*	816 (85)
No	80 (8)
Don't know/missing	59 (6)
Tuberculous infection	
Yes	493 (52)
TST result ≥10 mm	431 (45)
Previous history of TB/current TB disease	62 (6)
No	462 (48)

* Reported by verbal communication or based on proof of immunisation card.

IQR = interquartile range; BCG = bacille Calmette–Guérin; TST = tuberculin skin test.

**Figure 1.**
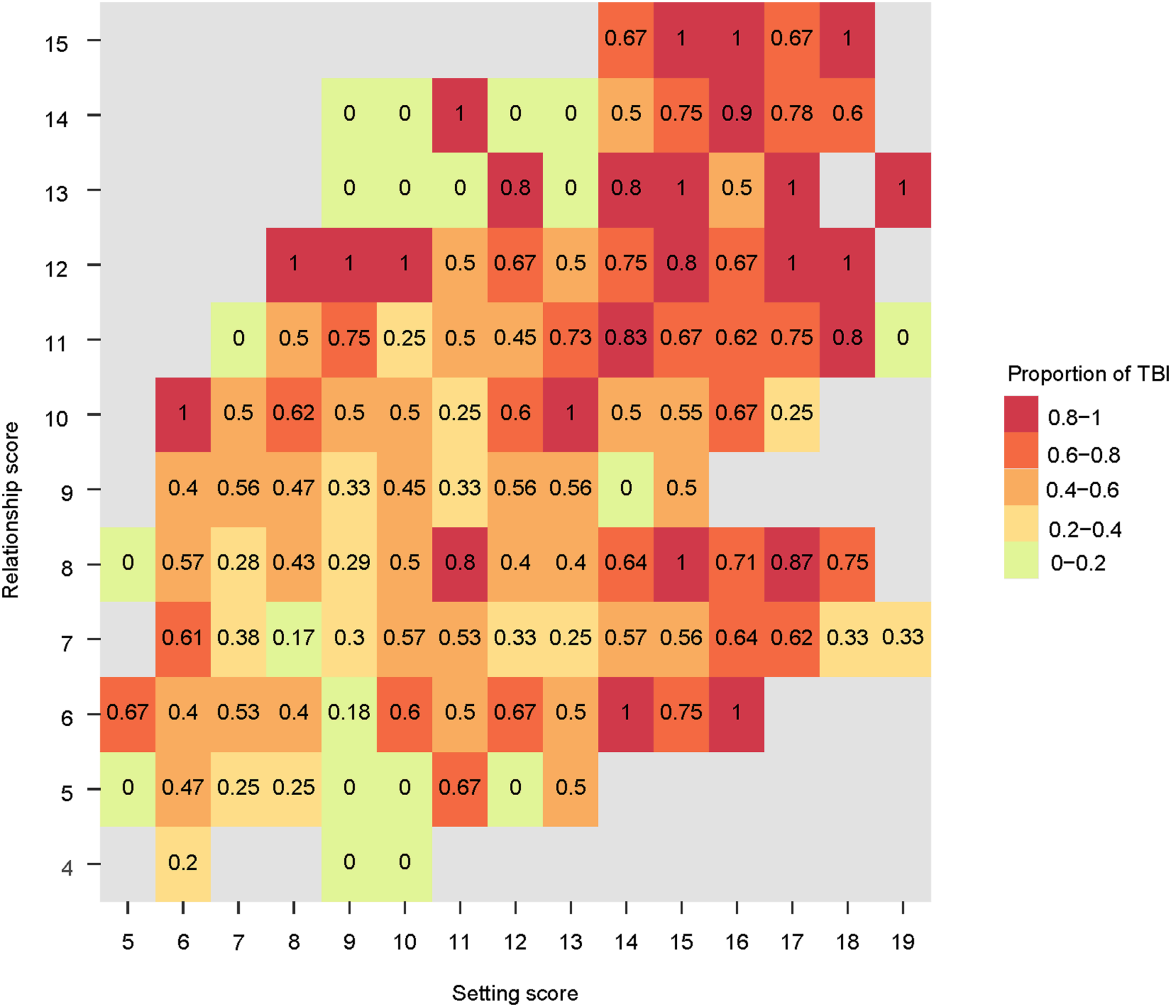
Proportion of TBI by setting and relationship scores. In this heatmap, the value in each cell of this heatmap represents the proportion of TBI among contacts with a given setting score and relationship score. TBI = tuberculous infection.

TBI prevalence in social networks was 52% (95% CI 48–55). Contacts who had setting scores ≥14 units and relationship scores ≥11 units had a higher TBI prevalence than other contacts (77% versus 49%, respectively; *P* < 0.0001) (Figure 1).

When we categorised contact scores into quartiles, we found a rise in the risk of infection with each increasing quartile. In very low, low, medium, and high setting-contact quartiles, TBI prevalence was 44%, 40%, 53% and 70%, respectively (Table 2, *P*_trend_ <0.0001). This corresponded to a crude PR of respectively 1.0 (95% CI 0.81–1.24), 1.30 (95% CI 1.04–1.63) and 1.64 (95% CI 1.33–2.01) for each quartile compared to the very low quartile. As for the relationship score, in very low, low, medium, and high-contact groups, TBI prevalence was respectively 41%, 47%, 53%, and 66% (*P*_trend_ <0.0001). This corresponded to a crude PR of respectively 1.13 (95% CI 0.93–1.37), 1.24 (95% CI 1.01–1.52) and 1.62 (95% CI 1.35–1.96) for each quartile compared to the very low quartile.

**Table 2.** Prevalence and crude PR (95% CI) for tuberculous infection among social contacts of TB cases by setting and relationship scores.

Category	Score range	*n*	Prevalence of tuberculous infection		Crude PR (95% CI)
			*n*	% (95% CI)	
Overall		955	493	52 (48–55)	
Setting score (continuous)	5.3–18.6	955			1.05 (1.03–1.07)
Setting score (categorical)					
Very low	5.3–7.0	239	105	44 (38–50)	1
Low	7.0–10.3	238	95	40 (34–46)	1.00 (0.81–1.24)
Medium	10.3–13.7	239	126	53 (46–59)	1.30 (1.04–1.63)
High	13.7–18.6	239	167	70 (64–76)	1.64 (1.33–2.01)
Relationship score (continuous)	4.0–14.8	955			1.08 (1.05–1.10)
Relationship score (categorical)					
Very low	4.0–6.4	242	99	41 (35–47)	1
Low	6.4–7.7	236	111	47 (41–53)	1.13 (0.93–1.37)
Medium	7.7–10.1	238	126	53 (46–59)	1.24 (1.01–1.52)
High	10.2–14.8	239	157	66 (60–72)	1.62 (1.35–1.96)

PR = prevalence ratio; CI = confidence interval.

The relationship between the 12 component variables of the two factors and TBI prevalence followed a similar pattern as the overall factor scores (Table 3). In the setting score, indoor meetings with reduced ventilation resulted in a higher prevalence of infection for contacts of index cases compared to outdoor meetings or rooms with ventilation. Similarly, the prevalence of infection was higher (74%) among contacts spending large portions of the week (>66.5 hours/week) with the case and individuals who shared meals daily with the index case (64%). Contacts who slept in the same room and/or same bed as the index case had a higher TBI prevalence (62–75%) than contacts who did not sleep in the same room as the index case (48%) (Table 3).

**Table 3.** Association between individual variables that comprised the setting and relationship domains and prevalence of TBI among contacts of TB cases.

Variable		TBI prevalence	
	*N* *	n*	%^†^ (95% CI)
Setting domain			
Nature of ventilation at usual meeting place			
Full ventilation	459	209	46 (41–50)
Fair ventilation	188	100	53 (46–60)
Minimal ventilation	154	100	65 (57–72)
Poor ventilation	154	84	55 (47–62)
Frequency of sleeping in the same room and bed			
Did not sleep in the same room, or bed	748	358	48 (44–51)
Slept in the same room, but not in the same bed	139	91	65 (57–73)
Slept in the same room and the same bed, not daily	16	12	75 (54–96)
Slept in the same room and the same bed, daily	52	32	62 (48–79)
Most recent meeting was indoors or outdoors			
Mostly meeting outdoors	452	201	44 (40–49)
Equally indoors/outdoors	251	132	53 (46–59)
Mostly meeting indoors	252	160	63 (58–69)
Frequency of sharing meals with contact			
Did not share meals	365	168	46 (41–51)
Shared meals, less than a day/week	83	32	39 (28–49)
Shared meals 1–3 days/week	134	62	46 (38–55)
Shared meals 4–6 days/week	59	31	52 (40–65)
Shared meals daily	314	200	64 (58–69)
Location of usual meeting with contact			
Outside home of TB case	415	49	49 (44–53)
Home of TB case	540	54	54 (50–58)
Frequency and duration of contact over the past month			
<3.5 h/week	276	107	39 (33–44)
3.5–28 h/week	378	187	49 (44–54)
28–66.5 h/week	216	136	63 (56–69)
>66.5 h/week	85	63	74 (65–83)
Relationship domain			
Case discussed and confided important life issues in the contact			
Did not discuss or confide	410	186	45 (40–50)
Discussed but did not confide	293	150	51 (45–57)
Discussed and confided	252	157	62 (56–68)
Case shared TB diagnosis with contact			
No	537	245	46 (41–50)
Yes	418	248	59 (55–64)
Frequency of care provided by contact in the past 3 months			
No care by contact	800	394	49 (46–53)
Care provided, less than a day/week	35	18	51 (35–68)
Provided care 1–3 days/week	41	25	61 (46–76)
Provided care 4–6 days/week	15	7	47 (21–72)
Provided care daily	64	49	76 (66–87)
Length of knowing the contact, years			
<2	375	164	44 (39–49)
2–4	165	83	50 (43–58)
5–6	190	109	57 (50–64)
>6	225	137	61 (54–67)
How well the case knows the contact			
Not well	15	1	7 (0–19)
Somewhat well	131	53	40 (32–49)
Moderately well	229	100	44 (37–50)
Very well	580	339	58 (54–62)
Means of transportation used most often with the contact			
None/walking	782	398	51 (47–54)
Another type of transportation	173	95	55 (47–62)

* *N* = the number of contacts in that category; *n* = the number of contacts with TBI.

† Proportion of contacts (%) with TBI.

TBI = tuberculous infection; CI = confidence interval.

For each variable in the relationship score, there was a monotonic increase in the proportion of infection as the extent of exposure increased within each category, except in care of contact (Table 3). The highest TBI prevalence occurred in contacts who discuss and confide with the case (62%), knew the TB diagnosis of the case (59%), provided daily care to the case (76%), and known each other for more than 6 years (61%) and was known very well by their index case (58%).

Since the nature of exposure may differ according to household or extra-household exposure (Supplementary Figure S2), we stratified the analysis by each category. Among household contacts, the majority had setting scores in the medium and high quartiles, and TBI prevalence was highest in the highest quartile (70%, *P*_trend_ <0.0001). Similarly, for the relationship score, TBI prevalence increased with increasing quartile in the contact score, from 41% in the very low quartile to 68% in the high quartile (*P*_trend_ = 0.0052). Among extra-household contacts, TBI prevalence tended to increase across quartiles from very low (44%), low (42%), medium (54%), to high (70%) (*P*_trend_ = 0.0713). For the relationship score, TBI prevalence in extra-household contacts was respectively 41%, 42%, 50 and 60% in the very low, low, middle, and high relationship-contact quartiles (*P*_trend_ = 0.0048).

Among contacts exposed to index cases with a high-smear grade, TBI prevalence increased from 42% in the lowest quartile of the setting score to 69% in the highest quartile (*P*_trend_ <0.0001) (Supplementary Figure S3). This pattern was not found among contacts of index cases with a low smear grade. Instead, TBI prevalence did not differ markedly among contacts in the lowest three quartiles (40%, 46%, and 32%, respectively) and was highest among contacts in the highest quartile of the setting score (75%) (*P*_trend_ = 0.0716). For the relationship score, both contacts of index cases with low or high smear grade results showed an increase in TBI prevalence according to the relationship quartiles (Supplementary Figure S3).

**Figure 2.**
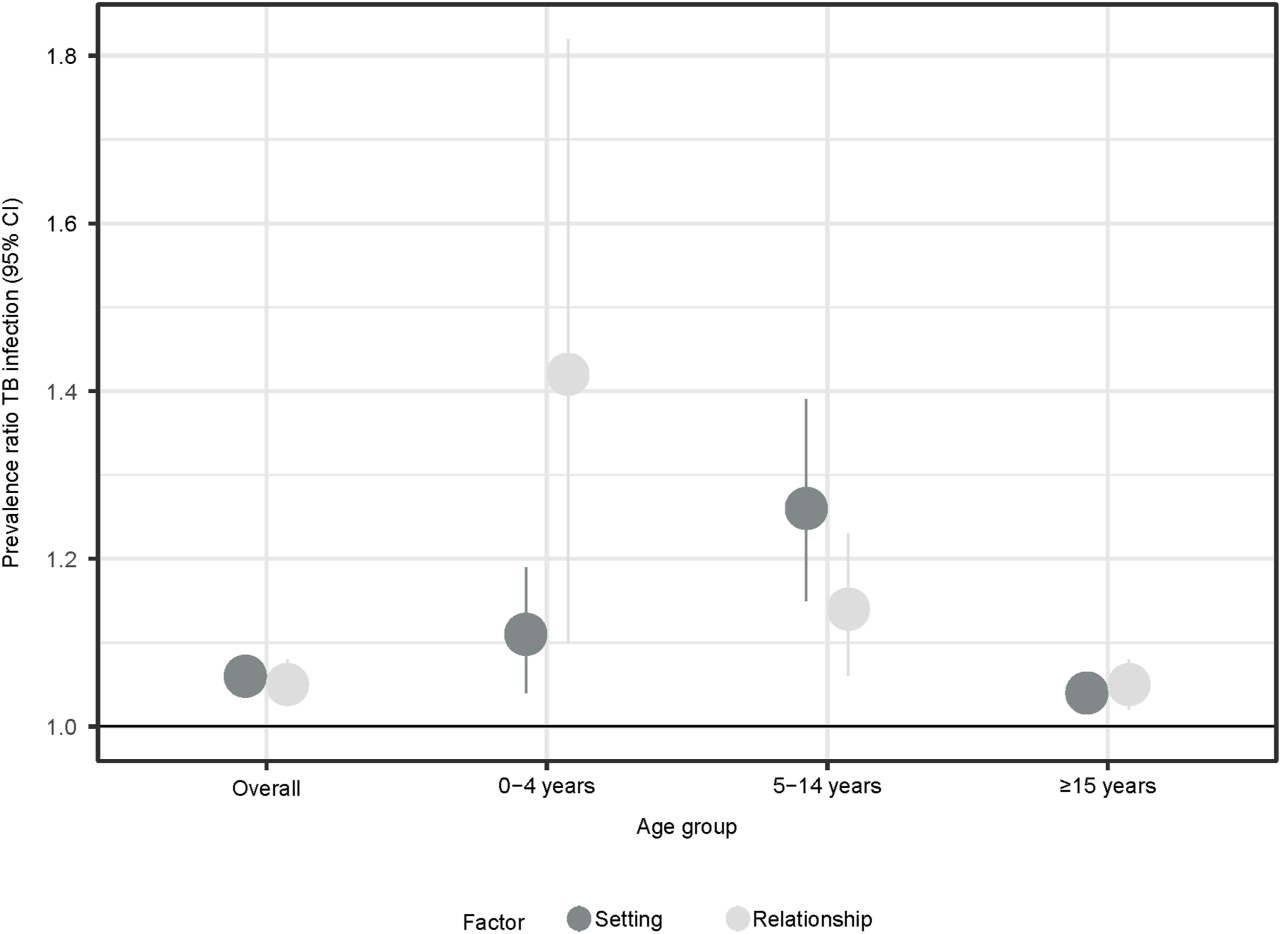
Adjusted prevalence ratios for the association between increasing scores in the setting and relationship scores and TBI, overall and stratified by age of contact. An adjusted prevalence ratio >1 indicates that for each increasing unit of the setting or relationship scores, there is a higher prevalence of TBI, after adjustment for other covariates (age, sex, HIV status, BCG vaccination status of contact; age, sex, HIV status, microscopy status and BMI of index case; knowledge of TB status of the contact by the index case and knowledge of cough status of the contact by the index case). TBI = TB infection; CI = confidence interval; BCG = bacilli Calmette-Guérin; BMI = body mass index.

After adjustment for covariates, the setting and relationship scores continued to be associated with TBI prevalence in contacts (Figure 2). This association was most pronounced in children. In the contacts 0–4 years old, the PR for infection was 1.11 (95% CI 1.04–1.19) for the setting score, meaning that with each increment of one unit in the score, the risk of infection was 11% higher. Similarly, for the relationship score, the PR for infection was 1.42 (95% CI 1.10–1.82). For contacts 5–14 years old, the adjusted PR was 1.26 (95% CI 1.15–1.39) for the setting score and 1.14 (95% CI 1.06–1.23) for the relationship score. Among adults, both scores were associated with infection, but the magnitude of the effect of the score was less. When analysed by quartiles, the results were similar to those of the crude PR. For the setting score, the adjusted PRs were 1.00 (95% CI 0.82–1.22), 1.33 (95% CI 1.07–1.65) and 1.81 (95% CI 1.47–2.23) for each quartile compared to the very low quartile. For the relationship score, these values were 1.10 (95% CI 0.91–1.34), 1.22 (95% CI 1.0–1.49) and 1.47 (95% CI 1.21–1.79).

## DISCUSSION

Ongoing transmission of *M. tuberculosis* is the central reason for the persistence of TB in the world today. Transmission of any pathogen is difficult to observe and measure, but defining adequate contact for transmission is more feasible. To this end, in a large observational study from an East African city with endemic TB, we developed a scoring method that used exploratory factor analysis as an agnostic approach to identify ways TB index cases interact with their social network contacts.^15^ The factor analysis identified two factors that described the setting and relationship between index cases and their contacts.

Here, we found that scores from each factor were consistently associated with TBI, especially in young children. We found consistent performance of the factor scores across different contexts, including differential exposure (household and extra-household) and infectiousness of the index case as measured by sputum microscopy. These integrated analyses provide strong support for the overall validity of the scores in assessing adequate contact for transmission.

The validity of the factor scores is further supported by the component variables that describe the settings of the interactions and the relationships between the cases and contacts. In prior studies, three component variables have been identified as risk factors for TBI in the setting factor.^28–30^ These variables included ventilation in the usual meeting place, sleeping with the index case, and interaction predominantly indoors or outdoors. For instance, it is known that higher ventilation rates decrease quanta concentration in inspired air, which in turn reduces the number of new incident cases,^31^ and prolonged and repeated exposures, as happens during sleep, are associated with new infections and diseases.^8,28,32^ Thus, including these variables in our setting domain supports its strong content validity.

Importantly, our setting-specific score disaggregated contacts with and without TBI in household and community contacts. For example, in community contacts, TBI prevalence was greatest (70%) among the quartile with the highest setting score. TBI prevalence was almost 30% lower in the lowest quartile (44%). Most transmission in high-burden settings occurs outside the household.^10^ However, efficient tools to identify high-risk individuals in the community are lacking. Potentially, an approach targeting community members with high setting or relationship scores may be viable to improve efficiency when screening the community and improve cost-effectiveness.

For the relationship factor, we found a strong association between the score and TBI in children under 15 years of age. This effect was especially pronounced among children under 5 years old because the component variables measure the close relationship with household members, especially their parents, who must care for them. The relationship domain likely reflected this intimate relationship and showed its utility in establishing *M. tuberculosis* transmission. Previous studies have used similar methods^8,33^ to measure exposure to TB and the risk of TBI and disease among child household contacts.^33^ Acuna-Villaorduna and colleagues modified this methodology to include adult household contacts in Brazil.^8^ Both studies found an association between the score and infection.

Our approach refines these approaches in important ways. First, we included household and extra-household contacts in developing the factors underlying the score, whereas earlier studies included only household contacts. Furthermore, we validated the performance of our scores separately for household and extra-household contacts. It has been established for a long time that the household is an environment for likely transmission of *M. tuberculosis*, especially to resident children. Our approach takes a step further to partially validate the use of the factors scores in the community.

Moreover, the results from these prior studies may not be directly comparable because they did not use social science methods to ascertain networks, and social networks are distinct from place to place.^34^ In addition, the background TB burden in Brazil is much lower than in Uganda, while South Africa has a higher prevalence of TBI and disease.^1^ Local transmission dynamics should be considered when evaluating such scores, and our methods should be validated in other settings.

Our study has limitations. First, we included contacts with latent and active TB in our definition of TBI. As this is a cross-sectional study, we cannot establish the directionality of the transmission for the latter category. Nevertheless, the large majority (87%) of our contacts had latent TB, suggesting it is unlikely that our findings would differ if we excluded contacts with active TB. Second, setting and relationship characteristics were reported by the index case, so recall bias is possible. Third, social mixing and behaviour are context-dependent;^24^ our findings might not be applicable in other settings.

Nevertheless, we developed the factors through an agnostic and unbiased approach to describe contact between the index case and social network members. We further validated the relationship between the factor scores and infection among contacts. As with any predictive tool, further development and testing should be done in other locations. This score was developed to quantitively measure one of the components that drives TB transmission, adequate contact.

Based on our analyses, we propose that the setting and relationship domains can contribute to characterising adequate contact among TB cases and their contacts. All infectious TB cases have a contact network that comprises both known (household members, friends, workmates) and unknown members with whom the index case may have a single contact. A previous study has suggested that causal contacts might represent more than 60% of the total contacts of the index cases,^35^ indicating that the household network is only partially defined through the social network.

## CONCLUSIONS

In conclusion, relationship and setting-specific grouped characteristics identified contacts with *M. tuberculosis* infection. These factor scores were especially apparent in children, a group at high risk of developing TB once infected. These factors may be useful in prioritising contact investigations of TB index cases, not only in the households of cases but also in the community.

## Supplementary Material



## References

[1] World Health Organization. Global tuberculosis report, 2019. Geneva, Switzerland: WHO, 2019.

[2] Whalen CC. The replacement principle of tuberculosis: why prevention matters. Am J Respir Crit Care Med. 2016;194(4):400–401. 27525460 10.1164/rccm.201603-0439ED PMC5003332

[3] Martinez L, Transmission of *Mycobacterium tuberculosis* in households and the community: a systematic review and meta-analysis. Am J Epidemiol. 2017;185(12):1327–1339. 28982226 10.1093/aje/kwx025 PMC6248487

[4] Saunders MJ, A household-level score to predict the risk of tuberculosis among contacts of patients with tuberculosis: a derivation and external validation prospective cohort study. Lancet Infect Dis. 2020;20(1):110–122. 31678031 10.1016/S1473-3099(19)30423-2 PMC6928575

[5] Tornee S, Risk factors for tuberculosis infection among household contacts in Bangkok, Thailand. Southeast Asian J Trop Med Public Health. 2004;35(2):375–383. 15691140

[6] Martinez L, Infectiousness of HIV-seropositive patients with tuberculosis in a high-burden African setting. Am J Respir Crit Care Med. 2016;194(9):1152–1163. 27181053 10.1164/rccm.201511-2146OC PMC5114446

[7] Morrison J, Pai M, Hopewell PC. Tuberculosis and latent tuberculosis infection in close contacts of people with pulmonary tuberculosis in low-income and middle-income countries: a systematic review and meta-analysis. Lancet Infect Dis. 2008;8(6):359–369. 18450516 10.1016/S1473-3099(08)70071-9

[8] Acuna-Villaorduna C, Intensity of exposure to pulmonary tuberculosis determines risk of tuberculosis infection and disease. Eur Respir J. 2018;51(1):1–9. 10.1183/13993003.01578-2017 PMC6719538 29348181

[9] Crampin A, Married to *M. tuberculosis*: risk of infection and disease in spouses of smear-positive tuberculosis patients. Trop Med Int Health. 2011;16(7):811–818. 21447058 10.1111/j.1365-3156.2011.02763.x PMC3378469

[10] Verver S, Proportion of tuberculosis transmission that takes place in households in a high-incidence area. Lancet. 2004;363(9404):212–214. 14738796 10.1016/S0140-6736(03)15332-9

[11] Kakaire R, Excess risk of tuberculous infection among extra-household contacts of tuberculosis cases in an African city. Clin Infect Dis. 2020;71(10):1–9. 33064142 10.1093/cid/ciaa1556 PMC8563168

[12] Chaves F, A longitudinal study of transmission of tuberculosis in a large prison population. Am J Respir Crit Care Med. 1997;155(2):719–725. 9032218 10.1164/ajrccm.155.2.9032218

[13] Kong L, Modeling heterogeneity in direct infectious disease transmission in a compartmental model. Int J Environ Res Public Health. 2016;13(3):253. 26927140 10.3390/ijerph13030253 PMC4808916

[14] McElroy PD, Outbreak of tuberculosis among homeless persons coinfected with human immunodeficiency virus. Clin Infect Dis. 2003;36(10):1305–1312. 12746777 10.1086/374836

[15] Castellanos ME, Defining adequate contact for transmission of *Mycobacterium tuberculosis* in an African urban environment. BMC Public Health. 2020;20(1):892. 32517672 10.1186/s12889-020-08998-7 PMC7285782

[16] Coutinho L, Scazufca M, Menezes PR. Methods for estimating prevalence ratios in cross-sectional studies. Rev Saude Publica. 2008;42(6):992–998. 19009156

[17] Spiegelman D, Hertzmark E. Easy SAS calculations for risk or prevalence ratios and differences. Am J Epidemiol. 2005;162(3):199–200. 15987728 10.1093/aje/kwi188

[18] Zou G. A modified Poisson regression approach to prospective studies with binary data. Am J Epidemiol. 2004;159(7):702–706. 15033648 10.1093/aje/kwh090

[19] Yelland LN, Salter AB, Ryan P. Performance of the modified Poisson regression approach for estimating relative risks from clustered prospective data. Am J Epidemiol. 2011;174(8):984–992. 21841157 10.1093/aje/kwr183

[20] Dheda K, Barry CE, Maartens G. Tuberculosis. Lancet. 2016;387(10024):1211–1226. 26377143 10.1016/S0140-6736(15)00151-8 PMC11268880

[21] Narasimhan P, Risk factors for tuberculosis. Pulm Med. 2013;2013:828939. 23476764 10.1155/2013/828939 PMC3583136

[22] Dodd PJ, Age- and sex-specific social contact patterns and incidence of *Mycobacterium tuberculosis* infection. Am J Epidemiol. 2016;183(2):156–166. 26646292 10.1093/aje/kwv160 PMC4706676

[23] Feenstra SG, A qualitative exploration of social contact patterns relevant to airborne infectious diseases in northwest Bangladesh. J Health Popul Nutr. 2013;31(4):424–430. 24592583 10.3329/jhpn.v31i4.19976 PMC3905636

[24] Mossong J, Social contacts and mixing patterns relevant to the spread of infectious diseases. PLoS Med. 2008;5(3):e74. 18366252 10.1371/journal.pmed.0050074 PMC2270306

[25] Kizza FN, Prevalence of latent tuberculosis infection and associated risk factors in an urban African setting. BMC Infect Dis. 2015;15(1):165. 25879423 10.1186/s12879-015-0904-1 PMC4392742

[26] Podewils LJ, Impact of malnutrition on clinical presentation, clinical course, and mortality in MDR-TB patients. Epidemiol Infect. 2011;139(1):113–120. 20429966 10.1017/S0950268810000907

[27] Nayak S, Acharjya B. Mantoux test and its interpretation. Indian Dermatol Online J. 2012;3(1):2–6. 23130251 10.4103/2229-5178.93479 PMC3481914

[28] Lienhardt C Risk factors for tuberculosis infection in sub-Saharan Africa: a contact study in The Gambia. Am J Respir Crit Care Med. 2003;168(4):448–455. 12773322 10.1164/rccm.200212-1483OC

[29] Rieder HL. Risk of travel-associated tuberculosis. Clin Infect Dis. 2001;33(8):1393–1396. 11565081 10.1086/323127

[30] Nardell EA. Indoor environmental control of tuberculosis and other airborne infections. Indoor Air. 2016;26(1):79–87. 26178270 10.1111/ina.12232

[31] Beggs CB, The transmission of tuberculosis in confined spaces: an analytical review of alternative epidemiological models. Int J Tuberc Lung Dis. 2003;7(11):1015–1026. 14598959

[32] Stevens H, Risk factors for tuberculosis in older children and adolescents: a matched case-control study in Recife, Brazil. Emerg Themes Epidemiol. 2014;11(1):20. 25642275 10.1186/s12982-014-0020-5 PMC4312596

[33] Mandalakas AM, Well-quantified tuberculosis exposure is a reliable surrogate measure of tuberculosis infection. Int J Tuberc Lung Dis. 2012;16(8):1033–1039. 22692027 10.5588/ijtld.12.0027 PMC11967560

[34] Horton KC, Systematic review and meta-analysis of sex differences in social contact patterns and implications for tuberculosis transmission and control. Emerg Infect Dis. 2020;26(5):910–919. 32310063 10.3201/eid2605.190574 PMC7181919

[35] McCreesh N, Comparison of indoor contact time data in Zambia and Western Cape, South Africa suggests targeting of interventions to reduce *Mycobacterium tuberculosis* transmission should be informed by local data. BMC Infect Dis. 2016;16(1):71. 26861444 10.1186/s12879-016-1406-5 PMC4746903

