# Supplementary material for: Performance of a score to characterise adequate contact among the social network of persons with TB
Source: IJTLD Open. 2024 Dec 1;1(12):556–63. doi: 10.5588/ijtldopen.24.0376 (PMC11636496; doi:10.5588/ijtldopen.24.0376)

http://dx.doi.org/10.5588/ijtldopen.24.0376

Performance of a score to characterise adequate contact among the social network of persons with TB

SUPPLEMENTARY DATA

Supplementary Figure S1. Flow diagram of study. 955 contacts out of 1006 contacts of 119 tuberculosis cases were evaluated regarding the association of the setting and relationship domains (exposures) with the presence or absence of tuberculous infection (outcome).

Supplementary Figure S2. Prevalence of tuberculous infection among contacts of tuberculosis cases, according to setting and relationship scores quartiles. Prevalence of tuberculosis (%) shown in overall population (top panel), household contacts (center panel) and extra-household contacts (bottom panel), according to setting and relationship score quartiles. Figure S3. Prevalence of tuberculous infection among contacts of tuberculosis cases, according to setting and relationship scores quartiles. Prevalence of tuberculosis (%) show stratified by smear result of the index case: 0 or 1 + (top panel), 2 or 3+ (bottom panel) and setting and relationship score quartiles.

Supplementary Figure S1. Flow diagram of study. 955 contacts out of 1006 contacts of 119 tuberculosis cases were evaluated regarding the association of the setting and relationship domains (exposures) with the presence or absence of tuberculous infection (outcome).

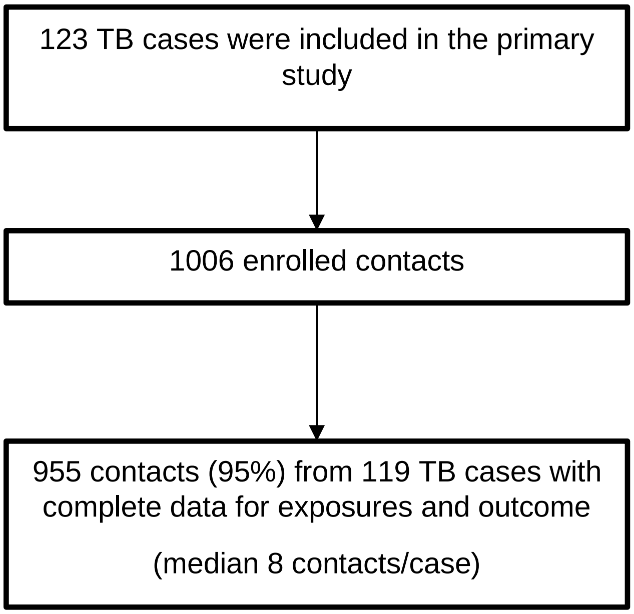

Supplementary Figure S2. Prevalence of tuberculous infection among contacts of tuberculosis cases, according to setting and relationship scores quartiles. Prevalence of tuberculosis (%) shown in overall population (top panel), household contacts (center panel) and extra-household contacts (bottom panel), according to setting and relationship score quartiles

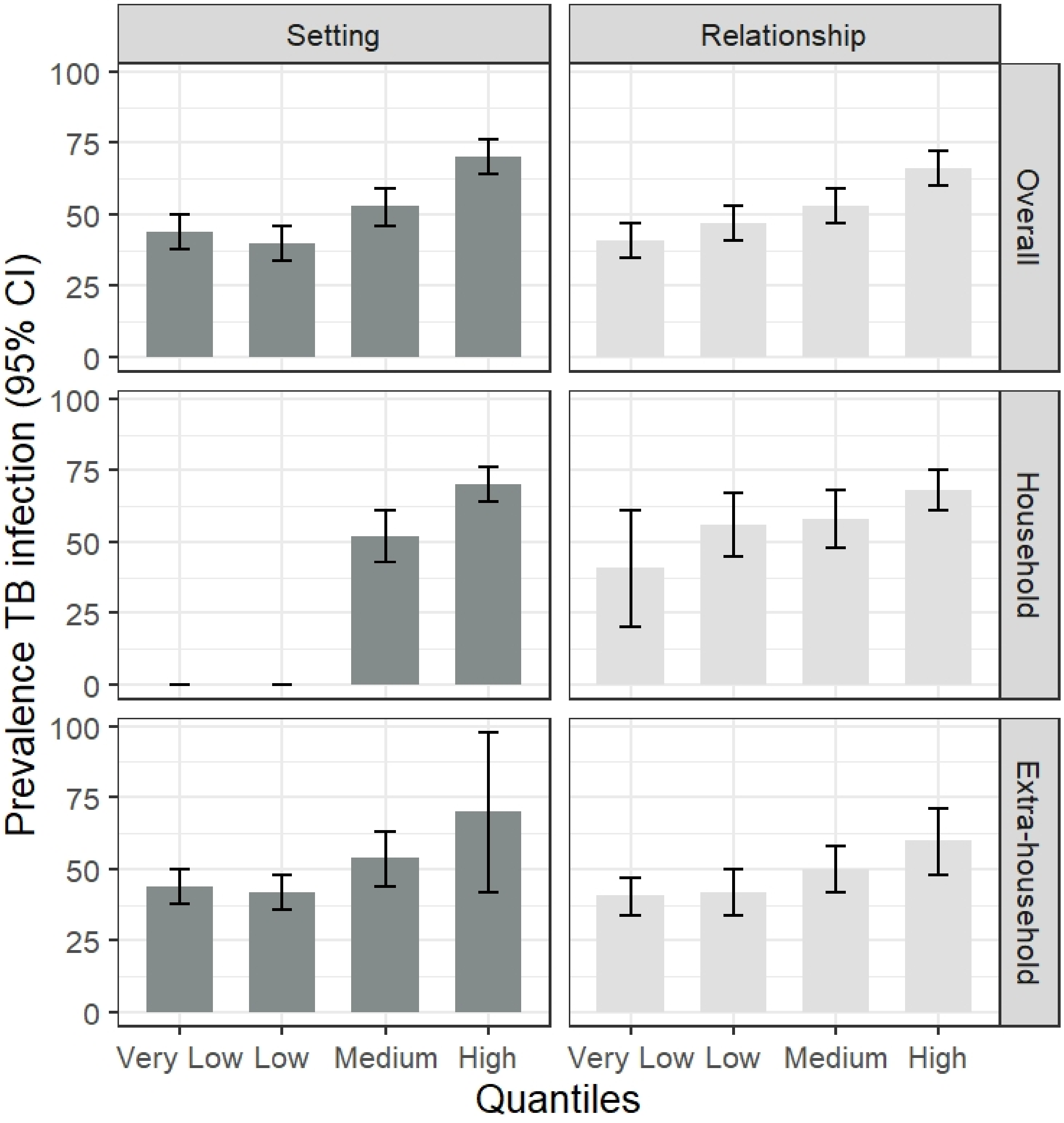

Supplementary Figure S3. Prevalence of tuberculous infection among contacts of tuberculosis cases, according to setting and relationship scores quartiles. Prevalence of tuberculosis (%) show stratified by smear result of the index case: 0 or 1+ (top panel), 2 or 3+ (bottom panel) and setting and relationship score quartiles.

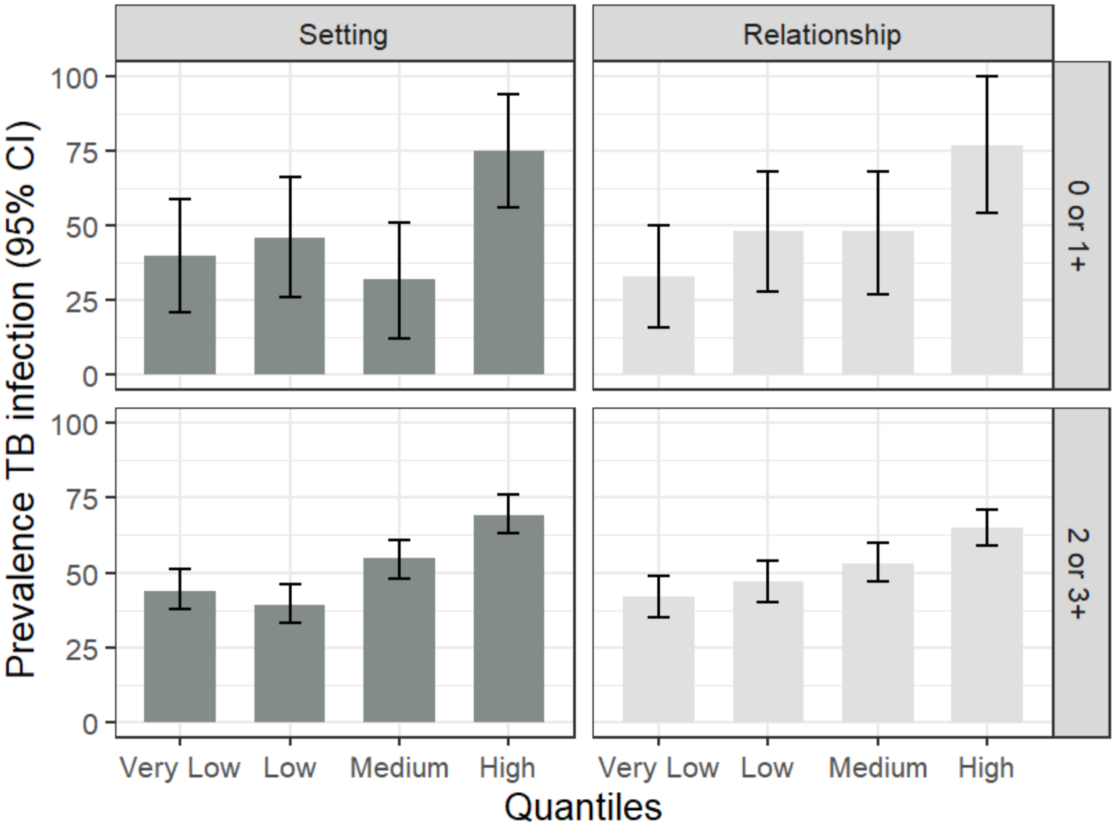

Supplement: Supplementary file 1 [file ijtldopen24-0376_supplementarydata1.pdf]
